# Supplementary material for: NCBO Ontology Recommender 2.0: an enhanced approach for biomedical ontology recommendation
Source: J Biomed Semantics. 2017 Jun 7;8:21. doi: 10.1186/s13326-017-0128-y (PMC5463318; doi:10.1186/s13326-017-0128-y)
Supplement: Supplementary file 1 — Ontology Recommender traffic summary. Summary of traffic received by the Ontology Recommender for the period 2014–2016, compared to the other most used BioPortal services. (PDF 27 kb) [file 13326_2017_128_MOESM1_ESM.pdf]

BioPortal API Traffic Summary[*]								
**Service**	**2014**		**2015**			**2016[**]**		
	**calls/mo[a]**	**%total[b]**	**calls/mo[a]**	**%total[b]**	**%var[c]**	**calls/mo[a]**	**%total[b]**	**%var[c]**
**Annotator**	2,390,392	22.83%	1,961,791	13.99%	-17.93%	484,795	2.58%	-75.29%
**Search**	820,073	7.83%	1,345,884	9.60%	64.12%	873,954	4.65%	-35.06%
**Mappings**	285,846	2.73%	502,298	3.58%	75.72%	213,003	1.13%	-57.59%
**Ontology Recommender**	**7,107**	**0.07%**	**6,359**	**0.05%**	**-10.53%**	**45,211**	**0.24%**	**611.00%**
**Resource Index**	2,388	0.02%	21,134	0.15%	785.20%	1,013	0.01%	-95.20%
**Other[d]**	6,962,751	66.51%	10,181,773	72.63%	46.23%	17,161,146	91.38%	68.55%
**TOTAL calls/mo[e]**	10,468,557	100.00%	14,019,239	100.00%	33.92%	18,779,122	100.00%	33.95%

[*] The calls made from the BioPortal website to the BioPortal API have been excluded.
[**] 2016 data are based on the period Jan-Oct (10 months).
[a] Mean number of API calls/month.
[b] Percentage of API calls/month with respect to the total number of BioPortal API calls/month.
[c] Percentage of variation with respect to the previous year.
[d] Other requests, which include browsing ontology classes (class details, paths to root, class tree, children, parents, etc.), ontologies (ontology details, root classes, groups, submissions, metrics, analytics, etc.), instances, projects, and users.
[e] Total number of API calls/month for the BioPortal API.

BioPortal Website Traffic Summary[*]								
**Webpage**	**2014**		**2015[**]**			**2016[***]**		
	**pv/mo[a]**	**%total[b]**	**pv/mo[a]**	**%total[b]**	**%var[c]**	**pv/mo[a]**	**%total[b]**	**%var[c]**
**Annotator**	3,371	0.79%	2,687	0.90%	-20.30%	3,048	0.99%	13.44%
**Search**	52,119	12.29%	56,905	18.96%	9.18%	72,951	23.77%	28.20%
**Mappings**	2,789	0.66%	7,213	2.40%	158.64%	2,258	0.74%	-68.70%
**Ontology Recommender**	**1,388**	**0.33%**	**925**	**0.31%**	**-33.38%**	**1,244**	**0.41%**	**34.55%**
**Resource Index**	1,715	0.40%	1,033	0.34%	-39.77%	1,154	0.38%	11.74%
**Other[d]**	362,637	85.52%	231,403	77.09%	-36.19%	226,229	73.72%	-2.24%
**TOTAL pv/mo[e]**	424,019	100.00%	300,166	100.00%	-29.21%	306,884	100.00%	2.24%

[*] Filtered traffic (excluding bots and AJAX requests).
[**] 2015 data are based on the periods Jan-Jul and Nov-Dec (9 months).
[***] 2016 data are based on the period Jan-Jul (7 months).
[a] Mean number of pageviews/month.
[b] Percentage of pageviews/month with respect to the total number of pageviews /month to the BioPortal website.
[c] Percentage of variation with respect to the previous year.
[d] Other pageviews, which include browsing ontology classes (class details, paths to root, class tree, children, parents, etc.), ontologies (ontology details, root classes, groups, submissions, metrics, analytics, etc.), instances, projects, and users.
[e] Total number of pageviews/month to the BioPortal website.
