# Supplementary material for: NCBO Ontology Recommender 2.0: an enhanced approach for biomedical ontology recommendation
Source: J Biomed Semantics. 2017 Jun 7;8:21. doi: 10.1186/s13326-017-0128-y (PMC5463318; doi:10.1186/s13326-017-0128-y)
Supplement: Supplementary file 2 — Default configuration settings. Default values used by the NCBO Ontology Recommender 2.0 for the parameters that control how the different scores are calculated, weighted and aggregated. (PDF 9 kb) [file 13326_2017_128_MOESM2_ESM.pdf]

Ontology Recommender 2.0 - Default configuration settings

<table>
<tr><th colspan="4">Ontology evaluation and score aggregation</th></tr>
<tr><th>Criterion</th><th>Weight</th><th colspan="2">Other parameters</th></tr>
<tr><td>Ontology coverage</td><td>0.55</td><td colspan="2">Preferred name (PREF) score: 10
Synonym (SYN) score: 5
Multi-word score: 3</td></tr>
<tr><td rowspan="2">Ontology acceptance</td><td rowspan="2">0.15</td><td>Presence</td><td>Weight (w_{visits}): 0.5
Repositories: UMLS</td></tr>
<tr><td>Visits</td><td>Weight ($w_{presence}$): 0.5
Repositories: BioPortal
Period: 12 months</td></tr>
<tr><td>Ontology detail</td><td>0.15</td><td colspan="2">Definitions threshold (k_d): 1
Synonyms threshold (k_s): 3
Properties threshold (k_p): 17</td></tr>
<tr><td>Ontology specialization</td><td>0.15</td><td colspan="2">N/A</td></tr>
<tr><th colspan="4">Ontology ranking</th></tr>
<tr><td colspan="2">Ranking size</td><td colspan="2">25</td></tr>
<tr><td colspan="2">Maximum number of ontologies/set</td><td colspan="2">3</td></tr>
</table>
